# Empathic concern for violators modulates victims’ evaluation of third-party punishment outcomes: behavioral and electroencephalography evidence

**DOI:** 10.1093/scan/nsag080

**Published:** 2026-09-25

**Authors:** Weihan Wang, Yinling Zhang, Rongrong Chen, Hao Zheng, Xiaoqin Mai

**Affiliations:** Department of Psychology, Renmin University of China, Beijing 100872, China; Research Center for Social Psychological Science and Engineering, Renmin University of China, Beijing 100872, China; School of Psychology, Shaanxi Normal University, Xi’an 710062, China; Department of Psychology, Renmin University of China, Beijing 100872, China; Department of Psychology, Renmin University of China, Beijing 100872, China; Department of Psychology, Renmin University of China, Beijing 100872, China; Research Center for Social Psychological Science and Engineering, Renmin University of China, Beijing 100872, China

**Keywords:** empathic concern, fairness, third-party punishment, outcome evaluation, EEG

## Abstract

When fairness conflicts with empathy-induced altruism, victims often reduce punishment to protect violators’ welfare. Whether this influence extends to outcome evaluations when punishment is implemented by third parties remains unclear. We combined an adapted Third-Party Dictator Game with electroencephalography to address this. Participants received unfair allocations from violators and evaluated third-party punishment (TPP) outcomes. Empathic concern was induced by portraying violators as being in need. Behaviorally, victims in the empathy (vs. non-empathy) condition rated no-punishment and partial-punishment outcomes as more appropriate but complete-punishment outcomes as less appropriate. Partial-punishment outcomes received the highest ratings in the empathy condition, reflecting a balance between protecting violators’ welfare and restoring fairness. Neurally, partial-punishment outcomes induced smaller P2 amplitudes in the empathy (vs. non-empathy) condition, suggesting lower motivational salience because they better matched victims’ goals. No-punishment outcomes induced larger late positive potential amplitudes in the empathy (vs. non-empathy) condition, reflecting sustained processing of their affective and motivational significance. Parietal alpha suppression was weaker in the empathy (vs. non-empathy) condition, indicating reduced sustained attention to TPP outcomes. Together, these findings suggest that empathic concern shapes victims’ evaluations of third-party norm-enforcement outcomes, extending its influence beyond contexts in which the empathizing individual also implements punishment.

## Introduction

Fairness is central to maintaining social order, typically enforced via punishment (Yang *et al*. 2024). However, violators often display suffering or remorse to elicit empathy (Tsoudis and Smith-Lovin 1998), creating a conflict between fairness and empathy-induced altruism. While research establishes that this conflict influences immediate punishment decisions (He *et al*. 2022, Zhang *et al*. 2025), it often overlooks subsequent outcome evaluation. Outcome evaluation is critical, reflecting subjective endorsement of decisions and providing feedback for behavioral adjustment (Yeung and Sanfey 2004). Moreover, previous studies often assume that empathizing and punishing are performed by the same agent. In reality, violators typically elicit empathy from victims, while punishment is implemented by non-involved third parties (Kanakogi *et al*. 2022). It remains unclear whether the fairness–altruism conflict persists in victims when punishment is implemented by third parties, and whether this conflict further extends to outcome evaluation. Accordingly, the present study aims to examine the influence of empathic concern for violators on victims’ evaluations of third-party punishment (TPP) outcomes.

### Victims’ evaluations of TPP outcomes

Evidence on outcome evaluation in punishment contexts is limited. Wu and Luo (2011) found that altruistic punishment outcomes maintaining social norms are evaluated more positively than no-punishment outcomes. Such punishment is considered altruistic because punishers incur costs to sanction violators and promote norm conformity (Fehr and Gächter 2002, Fehr and Fischbacher 2003), although its altruistic nature remains debated because it harms the violator without directly benefiting another person (Rodrigues *et al*. 2020). Most TPP studies have focused on evaluations of punishment behavior and show that bystanders evaluate punishment aimed at restoring fairness more favorably than no punishment (Raihani and Bshary 2015, Li *et al*. 2018). Unlike bystanders, victims are directly affected by unfairness and personally involved (Hofmann *et al*. 2018) and may therefore have a stronger desire to restore fairness. Appropriateness assesses whether punishment or sanctions are perceived as justified responses to norm violations, representing a key dimension of norm enforcement (Eriksson *et al*. 2017, 2021). Accordingly, the present study used appropriateness ratings as an index of outcome evaluation, and punishment outcomes were expected to be rated as more appropriate than no-punishment outcomes.

### Influence of empathic concern on victims’ evaluations of TPP outcomes

Empathy involves affective responses toward others and a tendency to adopt their perspective (Coke *et al*. 1978, Batson *et al*. 1988). As its affective component, empathic concern is an other-oriented emotional response elicited by and congruent with the perceived welfare of a person in need (Batson 1987, Batson *et al*. 1991). The empathy-altruism hypothesis proposes that empathic concern evokes altruism, which is defined as a motivational state with the ultimate goal of increasing another person’s welfare (Batson *et al*. 1988, 1991, 2007, Batson and Shaw 1991). This conceptualization is supported by behavioral and neuroscientific evidence (Davis 1983, Schroeder *et al*. 1988, Decety and Lamm 2009). Consequently, victims’ empathic concern toward violators may create a conflict between protecting violators’ welfare and upholding fairness.

This motivational conflict may first influence victims’ responses to the norm violation. Cognitively, empathic concern toward violators has been shown to increase victims’ perceived fairness of unfair allocations (He *et al*. 2022). Emotionally, although empathic concern toward victims has been shown to amplify moral outrage and increase punishment (Pfattheicher *et al*. 2019), empathic concern toward violators may instead attenuate moral outrage by considering their welfare. When victims subsequently evaluate TPP outcomes, their more lenient responses to the norm violation, together with their altruistic motivation to protect violators’ welfare, may make punishment outcomes seem less appropriate. Moreover, because empathic concern increases the perceived self-relevance of others’ outcomes (Liu *et al*. 2020), victims may perceive violators’ reduced payoffs as personally relevant, which may further reduce the perceived appropriateness of punishment outcomes. By contrast, no-punishment outcomes preserve violators’ payoffs and may therefore be perceived as more appropriate.

However, this does not mean that empathic concern eliminates victims’ motivation to restore fairness. Rather, altruism may temper this motivation while preserving the desire for fairness (Tan and Bolle 2006). Consistent with this view, empathic concern has been shown to reduce the intensity of TPP without eliminating punishment (Zhang *et al*. 2025). To distinguish whether empathic concern leads victims to prioritize the violator’s welfare over fairness or to balance these two motives, the present study distinguished between partial and complete punishment. No punishment preserves the violator’s welfare without restoring fairness, complete punishment restores fairness at the greatest cost to the violator, and partial punishment represents an intermediate action. Accordingly, if empathic concern promotes such a balance, victims should evaluate partial-punishment outcomes as more appropriate than both no-punishment and complete-punishment outcomes.

### Neural mechanisms underlying the influence of empathic concern on victims’ evaluations of TPP outcomes

Event-related potentials (ERPs) allow fine-grained examination of the neural dynamics of outcome evaluation, which involves an early semi-automatic stage reflecting motivational salience and a later stage reflecting elaborative evaluation (Leng and Zhou 2010). The early stage is indexed by the P2, a fronto-central positivity peaking around 200 ms. Larger P2 amplitudes are associated with prioritized processing of motivationally salient or unexpected outcomes, particularly those that exceed or fail to meet motivational predictions (Potts *et al*. 2006, San Martín *et al*. 2010, Wischnewski and Schutter 2018). P2 amplitudes during outcome evaluation are also sensitive to contextual factors (Peng *et al*. 2026), and empathic concern has been shown to modulate P2 amplitudes induced by unfair allocations during fairness-related decision-making (He *et al*. 2022). Because evaluating punishment outcomes involves assessing fairness restoration, empathic concern may modulate P2 responses to TPP outcomes. If empathic concern promotes a balance between fairness and altruism, partial-punishment outcomes should align most closely with victims’ motivational expectations and therefore induce smaller P2 amplitudes.

The later stage is indexed by the P3 and late positive potential (LPP). The P3 is a centro-parietal positivity peaking at 300–600 ms, associated with attentional allocation and affective evaluation (Gray *et al*. 2004, Polich 2007). It is sensitive to the valence, expectedness, and intensity of outcomes and feedback (Glazer and Nusslock 2022, Kirsch *et al*. 2022, Peters *et al*. 2024). However, existing evidence does not indicate that empathic concern modulates P3 amplitudes during outcome evaluation or fairness processing (Liu *et al*. 2020, He *et al*. 2022). Accordingly, the P3 was examined as an index to capture differences among outcomes.

The LPP is a positivity following the P3, with larger amplitudes reflecting sustained attention to the affective and motivational significance of outcomes (Hajcak *et al*. 2010, Glazer *et al*. 2018, Peters *et al*. 2024). Evidence from outcome evaluation showed that prosocial individuals exhibited larger LPP amplitudes for others’ gains than losses, suggesting that concern for others’ welfare modulated late-stage outcome processing (Hu *et al*. 2017). Complementary evidence showed that third parties’ empathic concern toward violators modulated offer-induced LPP amplitudes, with larger amplitudes reflecting sustained processing needed to resolve motivational trade-offs (Zhang *et al*. 2025). Accordingly, empathic concern may modulate LPP amplitudes during TPP outcome processing. Partial-punishment outcomes may best balance concern for violators’ welfare with fairness restoration, thereby requiring less sustained evaluation. By contrast, no-punishment and complete-punishment outcomes each favor one motive over the other, leaving the trade-off unresolved and requiring greater sustained attention.

Time–frequency analysis provides a complementary perspective on the neural dynamics underlying outcome evaluation. In the present study, these analyses were partly exploratory. Mid-frontal theta oscillations (4–7 Hz) reflect cognitive conflict detection and the signaling of control demands (Cohen and Donner 2013, Cavanagh and Frank 2014, Cohen 2014, Rodrigues *et al*. 2020). They are particularly sensitive to the primary and fundamental features of outcomes and feedback (Bernat *et al*. 2015, Bachman *et al*. 2022). Accordingly, theta oscillations were expected to primarily reflect the core features of TPP outcomes. Parieto-occipital alpha oscillations (8–13 Hz) are linked to attentional allocation and social-stimulus processing, with alpha suppression reflecting increased attentional engagement and neural arousal (Jensen *et al*. 2012, Song *et al*. 2024). Empathic concern has also been shown to modulate parieto-occipital alpha oscillations when participants made responder decisions on behalf of others (Chen *et al*. 2026a). Accordingly, alpha oscillations were expected to be modulated by empathic concern.

### Present study

The present study examined how empathic concern toward violators influences victims’ evaluations of TPP outcomes. We used an adapted Third-Party Dictator Game (TP-DG; Fehr and Fischbacher 2004). In the classic TP-DG, a proposer allocates resources between themself and a receiver, while an uninvolved third party observes the allocation and can reduce the proposer’s payoff as punishment. In the adapted paradigm, participants acted as receivers (i.e., victims of unfair allocations), whereas proposers who made unfair allocations served as violators. Participants evaluated outcomes resulting from three third-party actions: no punishment, partial punishment, and complete punishment. Notably, partial and complete punishment were forms of negative punishment, whereby the third party reduced the violator’s payoff.

Batson *et al*. (2007) suggested that empathic concern is more likely to arise when the target is perceived as being in need. Vignette-based manipulations have also been widely used to examine how prior information about interaction partners influences social decisions (Fouragnan *et al*. 2013, Stenzel *et al*. 2025). Accordingly, empathic concern was manipulated using vignette-based prior information, with proposers presented as either left-behind students in need (empathy condition) or regular students not in need (non-empathy condition) (He *et al*. 2022, Zhang *et al*. 2025). Victims’ appropriateness ratings and neural responses were compared across outcomes to examine how they weighed empathy-induced altruism against the motivation to uphold fairness. We proposed the following hypotheses:

**Hypothesis 1:** Regarding unfair allocations proposed by violators, victims would report higher fairness perceptions and lower moral outrage in the empathy (vs. non-empathy) condition.

**Hypothesis 2:** Regarding outcome evaluations, victims in the empathy (vs. non-empathy) condition would rate no-punishment and partial-punishment outcomes as more appropriate, but complete-punishment outcomes as less appropriate. Furthermore, partial-punishment outcomes would receive the highest appropriateness ratings in the empathy condition.

**Hypothesis 3:** Regarding ERP components, at the early semi-automatic stage, partial-punishment outcomes would induce smaller P2 amplitudes in the empathy (vs. non-empathy) condition. At the late stage, P3 amplitudes would primarily reflect differences among outcomes. For the LPP, no-punishment and complete-punishment outcomes would induce larger amplitudes in the empathy (vs. non-empathy) condition, whereas partial-punishment outcomes would show the opposite pattern.

**Hypothesis 4:** Regarding neural oscillations, frontal theta power would primarily differ across outcomes, whereas parieto-occipital alpha power would be primarily influenced by empathic concern.

## Materials and methods

### Participants

An a priori power analysis conducted using G*Power 3.1 indicated that a minimum of 19 participants was required to detect a medium-sized interaction effect in a 2 × 3 within-participants design (*f *= 0.25, α  =  0.05, power = 0.80; Faul *et al*. 2007). Previous research suggests that female participants generally exhibit higher levels of affective empathy and greater sensitivity to empathy-inducing contexts (Christov-Moore *et al*. 2014, Yan and Su 2018, Peng *et al*. 2021). Moreover, recruiting only female participants is relatively common in empathy-related research (Bierhoff and Rohmann 2004, Brown *et al*. 2021, Saulin *et al*. 2024, Chen *et al*. 2026a). To account for potential electroencephalography (EEG) data loss due to artifacts, 29 female university students (*M*_age_ = 22.45, *SD *= 2.14) were recruited. After excluding two participants because of excessive EEG artifacts, 27 participants remained in the final analysis (*M*_age_ = 22.33, *SD *= 2.15). All were right-handed, had normal or corrected-to-normal vision, and reported no neurological or psychiatric history. Participants provided written informed consent and received financial reimbursement. The study was approved by the local ethics review board.

### Experimental design and materials

The experiment employed a 2 (empathic concern: empathy, non-empathy) × 3 (third-party action: no punishment, partial punishment, complete punishment) within-participants design. In the adapted TP-DG, participants acted as receivers. In each round, they were instructed to assume that they and a proposer had contributed equally to a task and jointly earned 100 tokens. The proposer had full control over the allocation, whereas the receiver could not reject it. To elicit strong unfairness perceptions and capture the fairness–altruism conflict, the study focused on 90–10 allocations (±2 token jitter). Other allocations (50–50 to 80–20) served as fillers.

Participants were then informed that other participants acted as third parties. In each round, a third party observed the allocation and could spend tokens from a public resource pool to reduce the proposer’s payoff. This design was intended to reflect the costly nature of TPP without implying that third parties sacrificed their own tokens to intervene. For the 90–10 allocation, if the third party chose no punishment, the allocation remained unchanged. If the third party chose partial punishment, the proposer’s tokens were reduced by half of the initial difference (50–10). If the third party chose complete punishment, the proposer’s tokens were reduced to match the receiver’s, fully restoring fairness (10–10). Participants were told that third parties received a fixed payment regardless of their choices.

To minimize cognitive bias, roles and actions were described using neutral labels (Players A/B/C and maintain/partial reduction/complete reduction), and participants were informed that allocation and punishment decisions were made by different individuals across trials. Immediately after completing the experiment, participants were verbally asked by the experimenter whether they believed that the other players involved in the task were real and that the allocation and reduction decisions had been made by these players. Any indication of disbelief was recorded by the experimenter, and no participant expressed disbelief.

Empathic concern was manipulated by presenting proposers as either left-behind rural students or regular municipal students via text and images (He *et al*. 2022, Zhang *et al*. 2025). Participants were told that the proposers’ allocation decisions had been collected from students attending two schools, including a rural school for left-behind students and a regular municipal school. The left-behind rural students were described as experiencing financial hardship, poor school conditions, and limited educational opportunities, whereas the regular municipal students were described as having relatively better financial circumstances, school conditions, and educational opportunities. Manipulation check was conducted using the Empathic Response Questionnaire (ERQ; Batson *et al*. 2002, Pfattheicher *et al*. 2019, Zhang *et al*. 2025, Chen *et al*. 2026a, 2026b), in which participants rated sympathy, concern, and feeling moved on a 7-point scale. The three items were averaged to form an empathic concern index (Cronbach’s α = 0.97).

### Experimental procedure and measures

Stimuli were presented and behavioral data were recorded using E-Prime 3.0. As shown in Fig. 1, the avatars dressed in blue, white, and red represent the proposer, the receiver (i.e., the participant), and the third party, respectively. Each block began with proposer information designed to induce either an empathy or a non-empathy state. Participants then completed the ERQ as a manipulation check before proceeding to the formal trials. Each trial started with a fixation cross (600–800 ms), followed by the presentation of the proposer’s allocation for 1500 ms. After a variable interval (400–600 ms) and a 1000 ms blank screen, the outcome of the third party’s action was presented for 1500 ms. To reduce participants’ cognitive load, ratings were collected only in a subset of trials, in which participants rated either the perceived appropriateness of the final outcome (1 = very inappropriate, 7 = very appropriate) or their current level of anger after observing the outcome (1 = not at all angry, 7 = extremely angry). Thus, only one rating question was presented in each rating trial. These rating trials were randomly selected across formal and filler trials.

**Figure 1:**
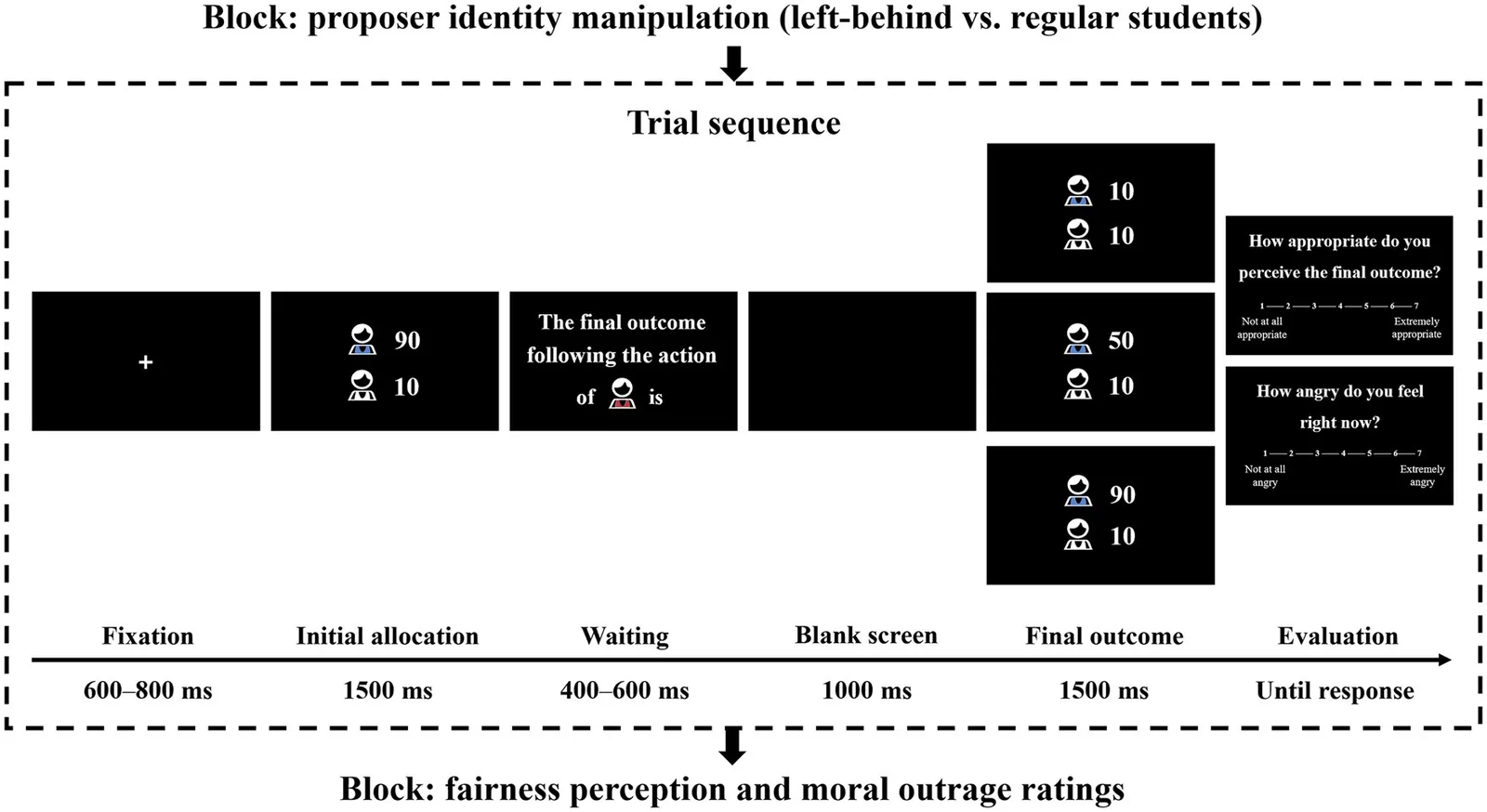
Overview of the experimental procedure. Empathic concern was manipulated at the start of each block via proposer information. In each trial, participants sequentially observed the proposer’s allocation and the final outcome resulting from the third party’s action. In a subset of trials, they then rated either the appropriateness of the final outcome or their current level of anger, with only one rating randomly presented per rating trial. At the end of each block, they additionally reported fairness perceptions and moral outrage in response to the proposer’s allocations.

At the end of each block, participants rated their fairness perceptions and moral outrage regarding the proposer’s allocations, including the 90–10 and filler allocations. Fairness perception was assessed with a single item (1 = very unfair, 7 = very fair), with higher scores indicating greater perceived fairness (Xie *et al*. 2022, Li *et al*. 2025, Chen *et al*. 2026b). Moral outrage was assessed using anger intensity (1 = not at all angry, 7 = very angry) and moral judgment (1 = strongly disagree, 7 = strongly agree), with the latter assessing the perceived immorality of the allocation. The two items were averaged to form the moral outrage index (Pfattheicher *et al*. 2019, Forbes *et al*. 2025). Block-level anger was distinct from trial-level anger, which reflected responses after TPP outcomes.

Punishment has been closely linked to state anger (Rodrigues *et al*. 2020), and previous research suggests that punishment may alleviate negative emotions elicited by norm violations (Wu and Luo 2011). Accordingly, we further examined whether anger elicited by the 90–10 allocation was reduced after observing TPP outcomes. We computed an anger reduction index as the difference between pre-outcome anger (assessed at the block level) and post-outcome anger (assessed trial by trial), with higher values indicating greater anger reduction. Trial-level anger ratings were primarily used to derive this index rather than analyzed as a dependent variable. Details on trials and ratings are provided in Supplementary Materials S1.

### EEG recording and data analysis

EEG was recorded from 64 channels (10–20 system) using a Neuroscan Synamps 2 amplifier (Compumedics, Abbotsford, Vic., Australia), referenced online to the left mastoid (1 kHz; impedances <5 kΩ). Offline analysis was conducted in MATLAB R2023b using the EEGLAB (version 2021.1; Delorme and Makeig 2004), ERPLAB (Lopez-Calderon and Luck 2014), and FieldTrip (Oostenveld *et al*. 2011) toolboxes. The data were first re-referenced to the average of the left and right mastoids. A 49–51 Hz notch filter and a 0.1–30 Hz band-pass filter were applied. Artifacts were corrected via independent component analysis (ICA), with artifactual components identified by the ICLabel plugin (Pion-Tonachini *et al*. 2019) and verified through manual inspection. Finally, trials containing voltage fluctuations exceeding ± 80 μV on any channel were excluded. For each participant, epochs were averaged within each of the six conditions, yielding 53 ± 1 trials per condition.

The time window for ERP analysis was 1000 ms, ranging from 200 ms before to 800 ms after the onset of the final outcome. A baseline correction was performed using the –200 ms pre-stimulus period. According to the purpose of our study, previous studies, and the grand average topographic maps, we measured the mean amplitude of P2 within the 170–250 ms time window at channel FCz (Chen *et al*. 2024); the mean amplitude of P3 within the 300–450 ms time window and LPP within the 450–700 ms time window at channel CPz (Hu *et al*. 2017, Glazer and Nusslock 2022).

Given the potential temporal overlap among ERP components, we additionally conducted a time-domain principal component analysis (PCA) using the Evoked ERP/EPO toolbox (Zhang *et al*. 2020). The first three components were identified as PCA_P2, PCA_P3, and PCA_LPP. Results were consistent with conventional ERP findings (see Supplementary Materials S2).

For the time-frequency analysis, EEG data were segmented into epochs from 1000 ms before to 1000 ms after the onset of the final outcome. The analysis was conducted using short-time Fourier transform (1–30 Hz, window width of 250 ms). To avoid boundary effects, the time interval from –800 to –200 ms was used for baseline correction. In line with prior studies (Bernat *et al*. 2015, Chen *et al*. 2024), we focused on frontal theta (4–7 Hz) and parietal alpha (8–13 Hz) oscillations. Theta power was analyzed at channel FC2 within the 130–260 ms window, and alpha power was analyzed at channel Pz within the 250–600 ms window.

All statistical analyses were performed using IBM SPSS Statistics (Version 27), with details on statistical criteria provided in Supplementary Materials S3. Ratings on the ERQ, fairness perception, and moral outrage were analyzed using paired-sample *t*-tests. Appropriateness ratings, anger reductions, ERP amplitudes, and neural oscillations were subjected to repeated-measures ANOVAs. Exploratory mediation analyses examined whether fairness perception and moral outrage accounted for the effect of empathic concern on evaluations of TPP outcomes; details are provided in Supplementary Materials S4.

## Results

### Behavioral results

#### Effectiveness of empathic concern manipulation

The ERQ scores were significantly higher in the empathy (*M* ± *SD*: 4.68 ± 1.32) than in the non-empathy condition (1.58 ± 0.80), *t*(26) = 13.05, *P* < .001, Cohen’s *d *= 2.51, confirming effective manipulation.

#### Fairness perception and moral outrage

For the 90–10 allocation (see Fig. 2a and b), participants reported significantly higher fairness perceptions in the empathy (2.16 ± 1.43) than in the non-empathy condition (1.15 ± 0.33), *t*(26) = 3.88, *P* = .001, Cohen’s *d *= 0.75. In contrast, moral outrage was significantly lower in the empathy (4.94 ± 1.83) than in the non-empathy condition (6.30 ± 0.86), *t*(26) = −5.05, *P* < .001, Cohen’s *d *= −0.97. Given that the difference scores for both variables deviated from normality, Wilcoxon signed-rank tests were also conducted. These tests similarly showed higher fairness perception in the empathy condition, *Z *= −3.52, *P* < .001, and lower moral outrage in the empathy condition, *Z *= −3.97, *P* < .001.

**Figure 2:**
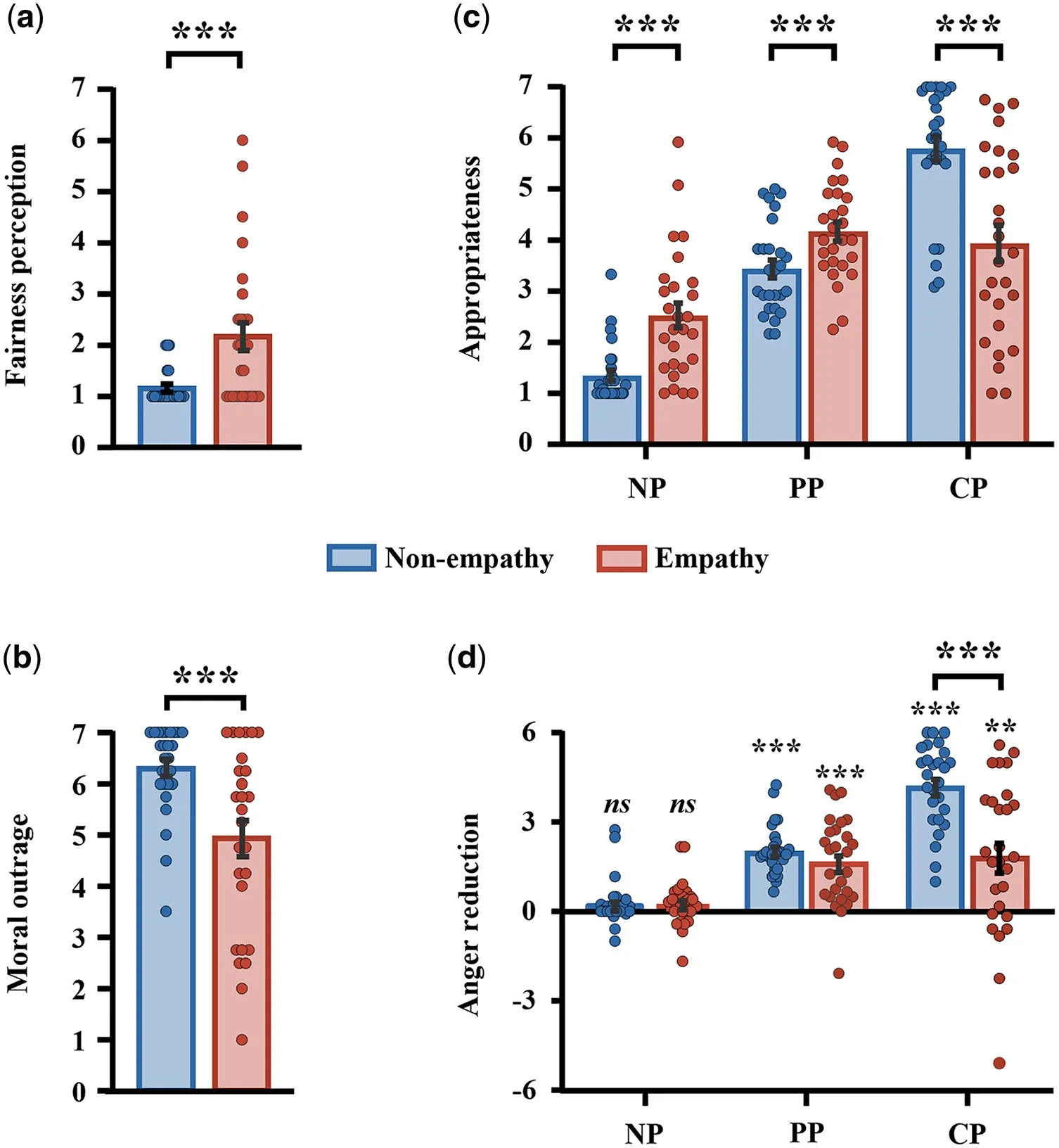
Behavioral evaluations. (a, b) Bar graphs showing the main effect of empathic concern on fairness perception (a) and moral outrage (b). (c, d) Bar graphs showing the interaction effect of empathic concern and third-party action (NP, no punishment; PP, partial punishment; CP, complete punishment) on appropriateness ratings (c) and anger reduction (d). Asterisks above bars indicate significance relative to zero. Each dot represents data from one participant. ***P < .001, **P < .01, ^ns^*P* > .10. Error bars indicate standard errors (SEM).

#### Appropriateness rating

The interaction between third-party action and empathic concern was significant (see Fig. 2c), *F*(1.57, 40.77) = 34.50, *P* < .001, η_p_^2^ = 0.57. Simple-effects analyses showed that appropriateness ratings were significantly higher in the empathy than in the non-empathy condition for no-punishment outcomes (2.53 ± 1.25 vs. 1.35 ± 0.57; *P* < .001, Cohen’s *d *= 1.05) and for partial-punishment outcomes (4.17 ± 0.93 vs. 3.44 ± 0.89; *P* < .001, Cohen’s *d *= 0.84). In contrast, for complete-punishment outcomes, appropriateness ratings were significantly lower in the empathy than in the non-empathy condition (3.94 ± 1.84 vs. 5.79 ± 1.25; *P* < .001, Cohen’s *d *= −0.99). The main effect of third-party action was significant (*F*(1.52, 39.60) = 55.35, *P* < .001, η_p_^2^ = 0.68), whereas the main effect of empathic concern was not significant (*P* = .859).

#### Anger reduction

The interaction between empathic concern and third-party action was significant (see Fig. 2d), *F*(1.34, 34.90) = 25.30, *P* < .001, η_p_^2^ = 0.49. Simple effects analysis showed that, for complete-punishment outcomes, participants reported a significantly smaller anger reduction in the empathy than in the non-empathy condition (1.87 ± 2.57 vs. 4.23 ± 1.39; *P* < .001, Cohen’s *d *= −0.99). By contrast, for no-punishment (0.26 ± 0.76 vs. 0.28 ± 0.77; *P* = .877) and partial-punishment outcomes (1.68 ± 1.46 vs. 2.05 ± 0.84; *P* = .167), anger reduction did not differ significantly between the empathy and non-empathy conditions. The main effects of empathic concern (*F*(1, 26) = 14.68, *P* < .001, η_p_^2^ = 0.36) and third-party action (*F*(1.44, 37.40) = 51.19, *P* < .001, η_p_^2^ = 0.66) were also significant. Because anger reduction was calculated as the difference between anger intensity (an item of moral outrage) and the anger rating after observing the outcome of the third party’s action, we repeated the analysis using rank-transformed anger-reduction scores as a sensitivity analysis. The results were consistent with the results reported above (see Supplementary Materials S5).

### ERP results

#### P2

The interaction between empathic concern and third-party action was significant (see Fig. 3), *F*(2, 52) = 3.35, *P* = .043, η_p_^2^ = 0.11. Simple effects analysis indicated that for partial-punishment outcomes, the P2 amplitudes were significantly smaller in the empathy (5.09 ± 2.76 μV) than in the non-empathy condition (5.96 ± 2.89 μV; *P* = .014, Cohen’s *d *= −0.51). However, no significant differences in P2 amplitudes between empathy and non-empathy conditions were found for either no-punishment outcomes (4.65 ± 2.76 μV vs. 4.41 ± 3.26 μV; *P* = .580) or complete-punishment outcomes (4.68 ± 2.95 μV vs. 4.53 ± 3.21 μV; *P* = .701). The main effect of third-party action was significant (*F*(2, 52) = 8.87, *P* < .001, η_p_^2^ = 0.25), whereas the main effect of empathic concern was not significant (*P* = .559).

**Figure 3:**
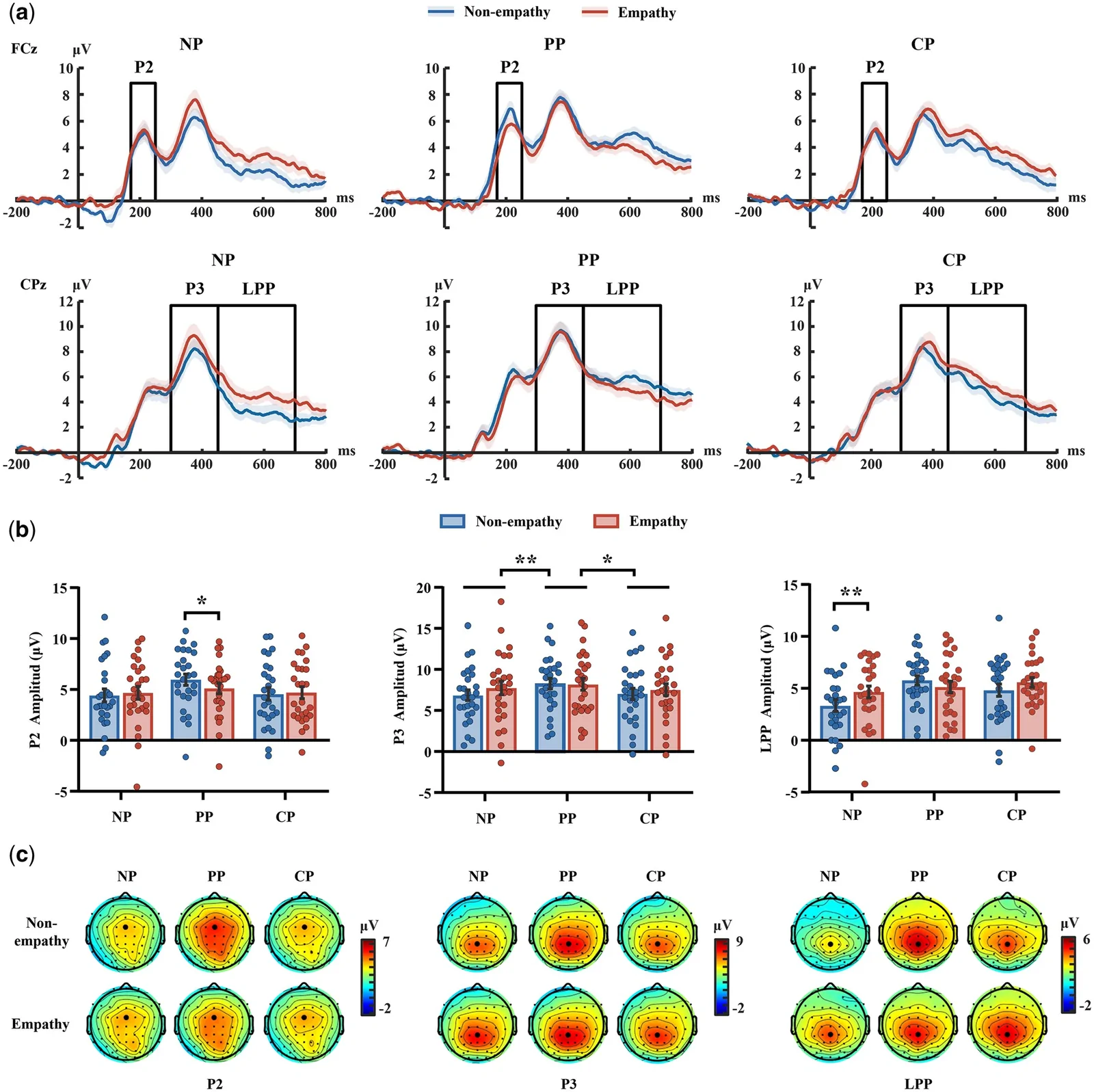
ERP results. (a) Grand-average ERP waveforms from the FCz and CPz electrodes. Black squares indicate the time window of the P2 amplitude (170–250 ms), P3 (300–450 ms), and LPP (450–700 ms). Shaded ribbons represent the standard error of the mean (SEM). (b) Bar graphs showing the mean amplitudes of P2, P3, and LPP for each condition. Scatter dots indicate all participants’ data. **P < .01, *P < .05. Error bars indicate SEM. (c) Scalp topographies among different conditions in the P2, P3, and LPP time windows. The electrodes included in the statistical analysis are highlighted. CP, complete punishment; NP, no punishment; PP, partial punishment.

#### P3

The main effect of third-party action was significant (see Fig. 3), *F*(2, 52) = 7.04, *P* = .002, η_p_^2^ = 0.21. Partial-punishment outcomes induced larger P3 amplitudes (8.22 ± 3.53 μV) than both no-punishment (7.29 ± 3.85 μV; *P* = .010, Cohen’s *d *= 0.62) and complete-punishment outcomes (7.27 ± 3.65 μV; *P* = .012, Cohen’s *d *= 0.61). No-punishment and complete-punishment outcomes did not differ (*P *= 1.000). Neither the main effect of empathic concern (*P* = .147) nor the interaction (*P* = .205) was significant.

#### LPP

The interaction between third-party action and empathic concern was significant (see Fig. 3), *F*(2, 52) = 6.93, *P* = .002, η_p_^2^ = 0.21. Further analyses showed that no-punishment outcomes induced significantly larger LPP amplitudes in the empathy condition than in the non-empathy condition (4.63 ± 3.02 μV vs. 3.33 ± 2.95 μV; *P* = .006, Cohen’s *d *= 0.57). By contrast, LPP amplitudes did not differ significantly between the empathy and non-empathy conditions for partial-punishment (5.13 ± 2.88 μV vs. 5.78 ± 2.36 μV; *P* = .072) and complete-punishment outcomes (5.57 ± 2.41 μV vs. 4.81 ± 3.10 μV; *P* = .075). The main effect of third-party action was significant (*F*(2, 52) = 12.43, *P* < .001, η_p_² = 0.32), whereas that of empathic concern was not (*P* = .071).

### Time-frequency results

#### Theta

The main effect of third-party action was significant (see Fig. 4), *F*(2, 52) = 3.25, *P* = .047, η_p_^2^ = 0.11. Theta power induced by partial-punishment outcomes (0.35 ± 0.42) was significantly higher than that induced by complete-punishment outcomes (0.21 ± 0.52; *P* = .051, Cohen’s *d *= 0.49). No significant differences were observed between no-punishment outcomes (0.28 ± 0.45) and either partial-punishment (*P* = .577) or complete-punishment outcomes (*P* = .704). Neither the main effect of empathic concern (*P* = .566) nor the interaction (*P* = .499) was significant.

**Figure 4:**
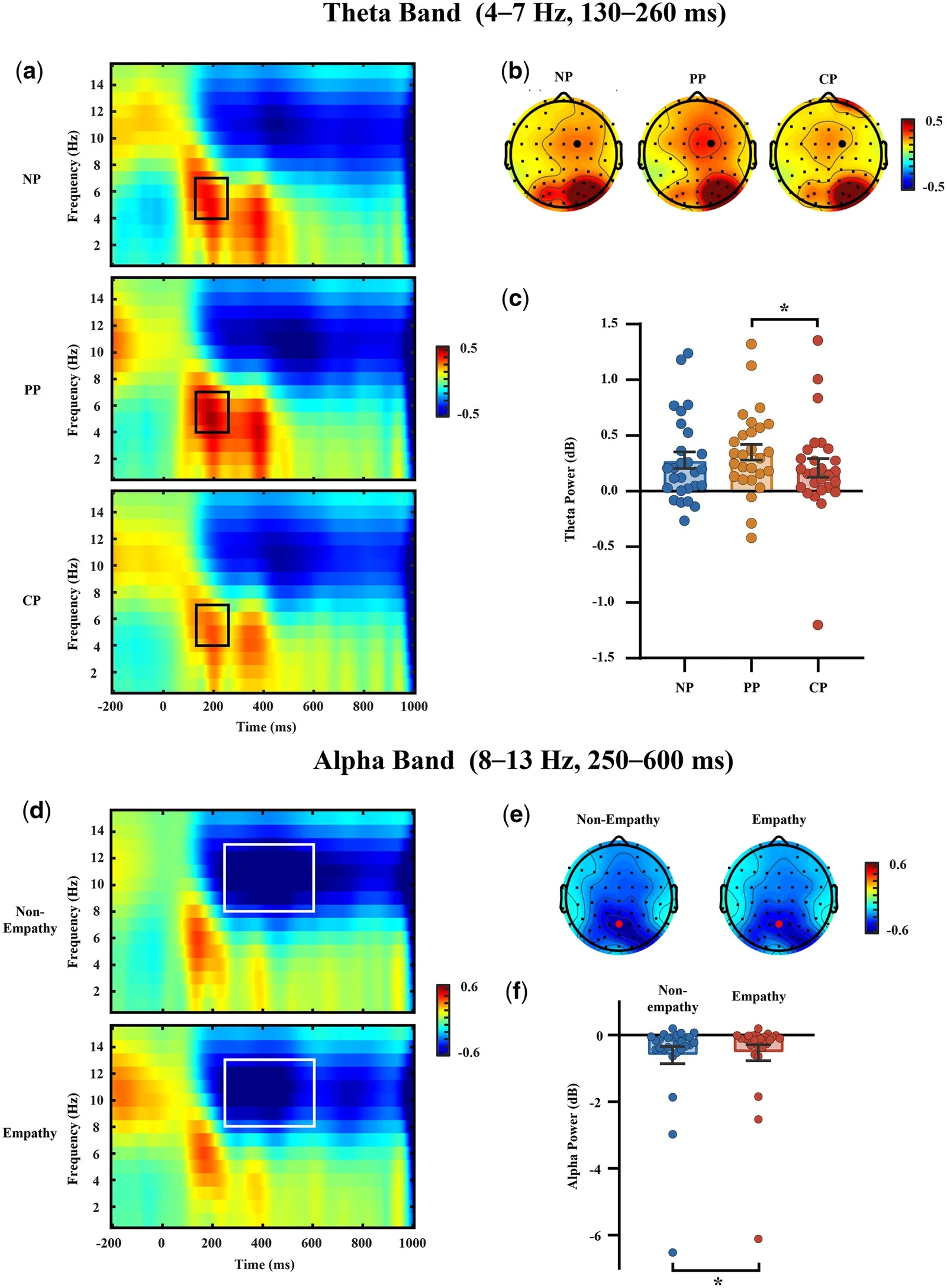
Time-frequency results. (a) Time-frequency spectrograms showing spectral power across time in the theta band (4–7 Hz, 130–260 ms), with analysis windows marked by squares. (b) Scalp topographies of theta power across conditions during the analysis time window. The electrodes included in the statistical analysis are highlighted. (c) Bar graphs showing the mean theta power for each condition. Scatter dots indicate all participants’ data. (d–f) Corresponding results in the alpha band (8–13 Hz, 250–600 ms). *P ≤ .05. Error bars indicate standard errors (SEM). CP, complete punishment; NP, no punishment; PP, partial punishment.

#### Alpha

The main effect of empathic concern was significant (see Fig. 4), *F*(1, 26) = 6.30, *P* = .020, η_p_^2^ = 0.20. Alpha power was significantly lower in the non-empathy condition (−0.59 ± 1.34) than in the empathy condition (−0.52 ± 1.31; *P* = .020, Cohen’s *d *= −0.48). Neither the main effect of third-party action (*P* = .271) nor the interaction (*P* = .261) was significant.

Given several relatively extreme participant-level values in the theta and alpha power data, we conducted two sensitivity analyses. First, robustness was assessed using 20% trimmed-mean methods. Second, values exceeding the mean ± 3 *SD* criterion for each condition and effect-specific within-participant difference scores were excluded, after which the original analyses were repeated. Full details and results are reported in Supplementary Materials S6.

## Discussion

This study examined how the conflict between empathy-induced altruism and the motivation to restore fairness influenced victims’ evaluations of TPP outcomes. Behaviorally, empathic concern increased fairness perceptions and reduced moral outrage toward violators’ 90–10 allocations. Regarding outcome evaluations, empathic concern increased appropriateness ratings for no-punishment and partial-punishment outcomes but decreased ratings for complete-punishment outcomes. EEG results demonstrated that at the semi-automatic stage, partial-punishment outcomes induced larger P2 amplitudes in the non-empathy condition than in the empathy condition. They also induced stronger frontal theta oscillations than complete-punishment outcomes. During late elaborative processing, partial-punishment outcomes likewise induced larger P3 amplitudes than no-punishment and complete-punishment outcomes, whereas no-punishment outcomes induced larger LPP amplitudes in the empathy condition than in the non-empathy condition. In addition, parietal alpha power was lower in the non-empathy condition than in the empathy condition, indicating stronger alpha suppression.

### Empathic concern influences victims’ evaluations of TPP outcomes

Regarding victims’ responses to violators’ 90–10 allocations, empathic concern increased fairness perceptions and reduced moral outrage. Distributive justice can incorporate need-based considerations rather than strict equality alone (Deutsch 1975), and fairness-related decisions in empathic contexts may place greater weight on the welfare of the empathized target (Chen *et al*. 2026a). Thus, perceiving violators as being in need may have led victims to incorporate their circumstances into fairness judgments, making the 90–10 allocation appear relatively less unfair. Moreover, because moral outrage is more likely when harmful outcomes are attributed to culpable intent (Ginther *et al*. 2022), empathic concern may have reduced the extent to which victims attributed the 90–10 allocation to blameworthy intentions, thereby lowering moral outrage.

Regarding victims’ evaluations of TPP outcomes, empathic concern increased appropriateness ratings for no-punishment and partial-punishment outcomes but decreased ratings for complete-punishment outcomes. This suggests that leniency toward disadvantaged violators extends to contexts where victims experience empathic concern while punishment is implemented by third parties (Ouyang *et al*. 2021, He *et al*. 2022, Zhang *et al*. 2025). No-punishment and partial-punishment outcomes may have been perceived as more appropriate because they avoided or limited losses to violators, whereas complete punishment imposed the greatest loss. Empathic concern may also have increased the perceived self-relevance of violators’ outcomes (Liu *et al*. 2020), making this loss more salient and reducing the perceived appropriateness of complete-punishment outcomes. Notably, partial-punishment outcomes received the highest appropriateness ratings in the empathy condition, suggesting that empathy-induced altruism did not override concern for fairness, as victims still expected some fairness restoration while limiting harm to violators.

Moreover, anger reduction following complete-punishment outcomes was smaller in the empathy condition than in the non-empathy condition. Given that state anger is an important driver of TPP (Rodrigues *et al*. 2020) and punishment can alleviate negative emotions (Wu and Luo 2011), greater initial anger in the non-empathy condition may have created a stronger need for severe punishment and thus greater anger relief. In the empathy condition, lower initial anger and concern for violators’ welfare may have reduced this need, leaving less anger to be alleviated.

### Empathic concern modulates the early semi-automatic stage of outcome evaluation

The early stage of outcome evaluation was indexed by P2 amplitudes and frontal theta oscillations. At this stage, empathic concern selectively modulated P2 responses to partial-punishment outcomes, with larger amplitudes in the non-empathy condition than in the empathy condition. In the non-empathy condition, partial-punishment outcomes may have been more motivationally salient because they appeared insufficient to correct the severe unfairness (San Martín *et al*. 2010, Wischnewski and Schutter 2018). However, in the empathy condition, restoring some fairness while limiting harm to violators may have aligned these outcomes more closely with victims’ motivational goals, thereby reducing their motivational salience. By contrast, no-punishment and complete-punishment outcomes provided clear signals of either no fairness restoration or full fairness restoration, and their early motivational salience may therefore have been relatively less sensitive to state empathic concern.

However, frontal theta activity primarily differentiated outcome types, with partial-punishment outcomes inducing stronger theta oscillations than complete-punishment outcomes. Given that theta oscillations are sensitive to primary feedback attributes (Bernat *et al*. 2015), this effect may reflect the rapid detection of conflicting features in partial-punishment outcomes, which reduced violators’ payoffs while leaving a payoff disparity, relative to the clearer fairness restoration signaled by complete-punishment outcomes. This interpretation should nevertheless be treated with caution, and the stability of the observed theta effect should be further evaluated in future studies with larger samples.

Overall, during the early semi-automatic stage, partial-punishment outcomes were rapidly processed because they combined a reduction in violators’ payoffs with residual inequality. However, in the empathy condition, these outcomes were more consistent with victims’ goal of balancing fairness and altruism, thereby reducing their motivational salience, consistent with their higher appropriateness ratings.

### Empathic concern modulates the late elaborative stage of outcome evaluation

The late elaborative stage of outcome evaluation was indexed by P3, LPP, and parietal alpha activity. Partial-punishment outcomes induced larger P3 amplitudes than no-punishment and complete-punishment outcomes, possibly reflecting the greater cognitive resources required to weigh their conflicting implications when judging their appropriateness (Hu *et al*. 2017, Glazer *et al*. 2018). Consistent with our hypothesis and previous findings, P3-related attentional allocation may be relatively insensitive to state empathy (Liu *et al*. 2020, He *et al*. 2022).

By contrast, the subsequent LPP was selectively modulated by empathic concern, with larger amplitudes for no-punishment outcomes in the empathy condition than in the non-empathy condition. The feedback-related LPP reflects sustained cognitive and attentional processing of an outcome’s affective value (Glazer *et al*. 2018). In the non-empathy condition, no-punishment outcomes primarily indicated that the unfairness of the 90–10 allocation remained uncorrected. However, in the empathy condition, they also meant that violators in need incurred no loss from punishment. Concern for violators’ welfare may therefore have increased the affective significance of these outcomes and prompted more sustained processing. In contrast, both partial-punishment and complete-punishment outcomes involved reductions in violators’ payoffs, which may themselves have been affectively salient during later processing.

A complementary explanation concerns unresolved motivational trade-offs. Larger LPP amplitudes have also been linked to the sustained processing required to resolve motivational trade-offs in punishment decisions (Zhang *et al*. 2025). In the empathy condition, no-punishment outcomes preserved violators’ welfare but failed to restore fairness, leaving the trade-off between altruistic and fairness-restoration motives unresolved. By contrast, in the non-empathy condition, victims’ evaluations were primarily guided by the motive to restore fairness, leaving no unresolved motivational trade-off and requiring less sustained processing.

Parietal alpha activity further revealed a broader effect of empathic concern across TPP outcomes. Alpha suppression was stronger in the non-empathy condition than in the empathy condition, suggesting that empathic concern reduced victims’ sustained attentional engagement with third parties’ responses to violators’ 90–10 allocations. This finding extends previous evidence that proposer-related information and empathic concern modulate parietal alpha activity during fairness-related decision-making (Chen *et al*. 2024, Chen *et al*. 2026a).

Taken together, the late-stage findings suggest that the conflicting features of partial-punishment outcomes may have continued to demand greater cognitive resources for evaluation. Empathic concern generally attenuated attentional engagement with TPP outcomes, while selectively enhancing the sustained processing of no-punishment outcomes, possibly because of their particular affective and motivational significance.

### Implications and limitations

Theoretically, the present study extends the influence of empathic concern beyond punishment decisions to subsequent outcome evaluations, even when punishment is implemented by third parties. Moreover, our findings show that the prioritization of empathy-induced altruism over fairness restoration is not absolute, suggesting that multiple motives in fairness-related decision-making may be integrated. Practically, these findings suggest that affective factors may shape judgments of punishment legitimacy, highlighting the importance of procedures that reduce emotional bias and promote consistent accountability.

Several limitations should be noted. First, all participants were female, limiting the generalizability of the findings to males. Previous research suggests that the relationship between empathy and norm-enforcement behavior may differ by gender. Specifically, Jin *et al*. (2024) found opposite associations between pain empathy and intervention tendencies in males and females, whereas a large-sample EEG study found no reliable gender differences in empathy-related neural responses (Pang *et al*. 2023). Because both studies examined pain empathy rather than the state empathic concern investigated here, whether gender modulates the present behavioral and neural effects remains unclear. Future studies should use gender-balanced samples to test whether the effects of empathic concern on broader fairness-related decisions differ by gender. Second, partial punishment was operationalized as a fixed amount, which limited the examination of punishment as a continuous gradient. Future research should incorporate more diverse punishment levels. Finally, empathic concern was induced only through proposer identity, which may not capture the full range of empathy elicitation. Future studies should employ multiple induction methods, such as narratives or facial expressions.

## Conclusion

When punishment is implemented by third parties, victims’ empathic concern toward violators does not lead them to disregard fairness norms. Instead, it encourages them to protect violators’ welfare while still valuing fairness restoration, as reflected in the highest appropriateness ratings and lower early motivational salience for partial-punishment outcomes. By contrast, no-punishment outcomes required more sustained processing because they protected violators’ welfare while leaving fairness unrestored. Overall, these findings indicate that empathic concern shapes victims’ motivational trade-offs between fairness and altruism in third-party norm-enforcement contexts.

## Supplementary Material

nsag080_Supplementary_Data

## Data Availability

All data supporting the findings of this study are publicly available on the Open Science Framework (OSF) at https://osf.io/j3b49/overview?view_only=7749c2895b784813b9deec4b010b028e.
