# Supplementary material for: Empathic concern for violators modulates victims’ evaluation of third-party punishment outcomes: behavioral and electroencephalography evidence
Source: Soc Cogn Affect Neurosci. 2026 Sep 25;21(1):nsag080. doi: 10.1093/scan/nsag080 (PMC13657424; doi:10.1093/scan/nsag080)
Supplement: Supplementary file 1 [file nsag080_supplementary_data.docx]

**Supplementary Materials for**

***Empathic Concern for Violators Modulates Victims’ Evaluation of Third-Party Punishment Outcomes: Behavioral and EEG Evidence***

# S1. Details of trials and ratings

All participants completed 12 practice trials prior to the formal task, followed by eight blocks (four in the empathy state and four in the non-empathy state), with block order randomized across participants. Each block consisted of 55 trials, including 45 formal trials (15 per third-party action) and 10 filler trials, with trial order randomized within each block. The filler set comprised three trials each for the 60–40, 70–30, and 80–20 allocations (with no punishment, partial punishment, and complete punishment each occurring once), and one trial for the 50–50 allocation (no punishment). In total, participants completed 440 trials, including 360 formal trials (60 per condition) and 80 filler trials. The task lasted approximately 60 minutes with self-paced breaks between blocks.

At the end of 18 formal trials and 3 filler trials per block, participants completed either an appropriateness rating or an anger rating, resulting in 144 formal trial-level ratings (72 appropriateness, 72 anger) and 24 filler ratings. In addition, fairness perception and moral outrage were assessed within each state. Each of the three items was administered twice for the 90–10 allocation, yielding six ratings per state. For filler allocations (50–50, 60–40, 70–30, 80–20), six ratings were randomly selected from the 12 possible ratings (three items × four allocations). These 12 ratings per state were randomly assigned to four blocks within each state, resulting in 24 block-level ratings in total.

# S2. Time-domain PCA results

Ten principal components were extracted, explaining 90% of the total variance. Based on their respective temporal and spatial distributions, the first three components were identified as PCA_P2, PCA_P3, and PCA_LPP. We analyzed mean amplitudes of PCA_P2 at FCz within 140–220 ms, and mean amplitudes of PCA_P3 and PCA_LPP at CPz within 220–400 ms and 450–700 ms, respectively. The results were as follows.

***PCA_P2*.** The interaction between empathic concern and third-party action was significant, *F*(2, 52) = 4.54, *p* = .015, η_p_^2^ = 0.15. Simple effects analysis revealed that for partial-punishment outcomes (see Fig. S1), the PCA_P2 amplitudes were significantly larger in the non-empathy (2.79 ± 3.11 μV) than in the empathy condition (1.85 ± 3.00 μV; *p* = .006, Cohen’s *d* = 0.58). However, for both no-punishment (1.64 ± 3.01 μV vs. 1.89 ± 3.04 μV; *p* = .485) and complete-punishment outcomes (1.92 ± 3.05 μV vs. 1.84 ± 2.92 μV; *p* = .801), there were no significant differences between the two conditions. The main effect of third-party action was significant (*F*(2, 52) = 3.68, *p* = .032, η_p_^2^ = 0.12), whereas the main effect of empathic concern was not significant (*p* = .264).

***PCA_P3.*** The main effect of third-party action was significant (see Fig. S1), *F*(2, 52) = 6.55, *p* = .003, η_p_^2^ = 0.20. The PCA_P3 amplitudes for partial-punishment outcomes (1.63 ± 1.83 μV) was significantly larger than that for no-punishment (1.13 ± 2.11 μV; *p* = .009, Cohen’s *d* = 0.63) and complete-punishment outcomes (1.17 ± 1.96 μV; *p* = .033, Cohen’s *d* = 0.53), with no significant difference between no-punishment and complete-punishment outcomes (*p* = 1.00). The main effect of empathic concern (*p* = .318) and the interaction between empathic concern and third-party action (*p* = .288) were not significant.

***PCA_LPP.*** The interaction between empathic concern and third-party action was also significant (see Fig. S1), *F*(2, 52) = 7.77, *p* = .001, η_p_^2^ = 0.23. Simple-effects analyses showed that no-punishment outcomes induced significantly larger PCA_LPP amplitudes in the empathy condition (1.06 ± 1.69 μV) than in the non-empathy condition (0.28 ± 1.77 μV; *p* = .005, Cohen’s *d* = 0.37). By contrast, PCA_LPP amplitudes did not differ significantly between the empathy and non-empathy conditions for complete-punishment outcomes (1.55 ± 1.39 μV vs. 1.10 ± 1.75 μV; *p* = .069) and for partial-punishment outcomes (1.38 ± 1.70 μV vs. 1.80 ± 1.36 μV; *p* = .053). The main effect of third-party action was significant (*F*(2, 52) = 11.46, *p* < .001, η_p_² = 0.31), whereas the main effect of empathic concern was not significant (*p* = .079).

**Fig. S1.** **PCA results. a**, Grand-average waveforms from the FCz and CPz electrodes. Black squares indicate the time window of the PCA_P2 amplitude (140–220 ms), PCA_P3 (220–400 ms), and PCA_LPP (450–700 ms). **b**, Bar graphs showing the mean amplitudes of PCA_P2, PCA_P3, and PCA_LPP for each condition. Scatter dots indicate all participants’ data. ***p* < 0.01, **p* < 0.05. Error bars indicate standard errors (SEM). **c**, Scalp topographies among different conditions in the PCA_P2, PCA_P3, and PCA_LPP time windows. The electrodes included in the statistical analysis are highlighted. *Note.* NP = no punishment; PP = partial punishment; CP = complete punishment.

# S3. Statistical criteria and parameters

All statistical analyses in the present study were performed using IBM SPSS Statistics software (Version 27; SPSS Inc., IBM, Chicago, IL, USA). Sphericity was assessed with Mauchly’s test; when violated, degrees of freedom were adjusted using the Greenhouse–Geisser correction. Post hoc comparisons used Bonferroni adjustments to *p* values. Mediation analyses were conducted using the path coefficient method (Model 6) implemented in the SPSS PROCESS (v4.1) macro (Hayes, 2018). The significance of indirect effects was evaluated using a nonparametric bootstrap procedure with 5,000 resamples and a 95% confidence interval (CI). An indirect effect was considered statistically significant if the CI did not include zero.

# S4. Exploratory mediation analyses of how empathic concern influences evaluations of TPP outcomes

## S4.1 Rationale and data analysis

To explore the possible psychological mechanisms through which empathic concern influenced victims’ evaluations of TPP outcomes, we conducted separate mediation analyses for appropriateness ratings of no-punishment, partial-punishment, and complete-punishment outcomes. Because social decisions are jointly shaped by cognitive and emotional processes (Evans, 2003; Strack & Deutsch, 2004), and cognitive appraisals can exert top-down modulation over emotional responses (Evans & Stanovich, 2013; Feng et al., 2015; Ochsner & Gross, 2005; Ochsner et al., 2009), fairness perception was specified as the first mediator, followed by an emotional response. Moral outrage elicited by the unfair allocation was examined as the second mediator in all three models.

However, the serial indirect effect through fairness perception and moral outrage was not significant for complete-punishment outcomes, 95% CI [−0.64, 0.04]. Given that empathic concern significantly influenced anger reduction following complete punishment, an additional serial mediation model was tested with fairness perception and anger reduction as sequential mediators.

## S4.2 Results

For no-punishment outcomes (see Fig. S2a), the regression model was significant, *R*^2^ = 0.80, *F*(3, 50) = 67.39, *p* < .001. Empathic concern positively predicted fairness perception (*β* = 0.88, *p* < .001), which negatively predicted moral outrage (*β* = −0.75, *p* < .001), and moral outrage further negatively predicted appropriateness ratings for no-punishment outcomes (*β* = −0.25, *p* = .022). Meanwhile, empathic concern exerted a significant direct effect on these ratings (*β* = 0.29, *p* = .044). Bootstrap testing indicated a significant serial mediation effect (effect size = 0.17, 95% CI [0.01, 0.43]).

For partial-punishment outcomes (see Fig. S2b), the regression model was significant, *R*^2^ = 0.24, *F*(3, 50) = 5.38, *p* = .003. Empathic concern positively predicted fairness perception (*β* = 0.88, *p* < .001), which negatively predicted moral outrage (*β* = −0.75, *p* < .001), and moral outrage negatively predicted appropriateness ratings for partial punishment (*β* = −0.43, *p* = .041). Meanwhile, empathic concern had a significant direct effect on these ratings (*β* = 0.85, *p* = .003). Bootstrap testing showed a significant serial mediation effect (effect size = 0.29, 95% CI [0.05, 0.73]).

For complete-punishment outcomes, the additional serial mediation model with fairness perception and anger reduction as sequential mediators is shown in Fig. S2c. The overall model was significant (*R*^2^ = 0.58, *F*(3, 50) = 23.33, *p* < .001). Empathic concern positively predicted fairness perception (*β* = 0.88, *p* < .001), which negatively predicted anger reduction (*β* = −0.69, *p* < .001), and anger reduction positively predicted appropriateness ratings for complete punishment (*β* = 0.74, *p* < .001). Meanwhile, the direct effect of empathic concern on punishment preference was not significant (*β* = −0.39, *p* = .069, 95% CI [−1.48, 0.06]). Bootstrap testing indicated a significant serial mediation effect (effect size = −0.45, 95% CI [−0.80, −0.17]).

**Fig. S2. Mediation analysis results.** The indirect effect of empathic concern on appropriateness ratings, mediated by fairness perception and moral outrage for no-punishment outcomes (a) and partial-punishment outcomes (b), and by fairness perception and anger reduction for complete-punishment outcomes (c). ****p* < 0.001, ***p* < 0.01, **p* < 0.05. *Note*. NP = no-punishment; PP = partial-punishment; CP = complete-punishment.

## S4.3 Interpretation

The exploratory mediation analyses provided a possible account of how empathic concern influenced evaluations of TPP outcomes. For no-punishment and partial-punishment outcomes, empathic concern was associated with higher appropriateness ratings through increased fairness perception and reduced moral outrage. Because moral outrage depends partly on appraisals of culpable intent and harm (Ginther et al., 2022), perceiving the violator’s 90–10 allocation as relatively fairer may have reduced the perceived blameworthiness of the violation, thereby weakening moral outrage. This interpretation is consistent with previous research on second- and third-party punishment, showing that lower fairness perceptions elicit stronger anger responses, which in turn promote punishment (Mendoza et al., 2014; Zhang et al., 2023). Accordingly, weaker moral outrage may have reduced victims’ punitive motivation, making no-punishment and partial-punishment outcomes appear more proportionate and appropriate.

For complete-punishment outcomes, empathic concern was associated with lower appropriateness ratings through increased fairness perception and reduced anger relief following complete punishment. Because the proposer’s 90–10 allocation elicited less initial anger and a weaker need for severe punishment in the empathy condition, the extent to which punishment relieved victims’ negative emotions was also reduced (Wu & Luo, 2011). Given that affective changes can inform evaluative judgments (Schwarz & Clore, 1983), this smaller reduction in anger may have contributed to lower appropriateness ratings. As these analyses were exploratory, these pathways should be interpreted as possible explanations rather than established psychological mechanisms.

# S5. Sensitivity analysis for anger reduction

Because anger reduction was calculated as the difference between anger intensity (an item of moral outrage) and the anger rating after observing the outcome of the third party’s action, we further examined whether the results were robust to non-normality. Shapiro-Wilk tests showed that, in the non-empathy condition, anger-reduction scores deviated from normality after no punishment (*W* = 0.68, *p* < .001), partial punishment (*W* = 0.92, *p* = .047), and complete punishment (*W* = 0.83, *p* < .001). In the empathy condition, anger-reduction scores did not significantly deviate from normality after no punishment (*W* = 0.93, *p* = .053), partial punishment (*W* = 0.98, *p* = .912), or complete punishment (*W* = 0.94, *p* = .134). Therefore, all anger-reduction scores across the six conditions were rank-transformed, and the same 2 (empathic concern: empathy, non-empathy) × 3 (third-party action: no punishment, partial punishment, complete punishment) repeated-measures ANOVA was conducted on the rank-transformed scores.

The results were consistent with those based on the original anger-reduction scores. The interaction between empathic concern and third-party action remained significant, *F*(1.48, 38.42) = 27.65, *p* < .001, η_p_² = 0.52. Further analysis showed that, for complete-punishment outcomes, anger reduction was significantly smaller in the empathy condition than in the non-empathy condition (*p* < .001). By contrast, anger reduction did not differ significantly between the empathy and non-empathy conditions for no-punishment outcomes (*p* = .571) and partial-punishment outcomes (*p* = .150). The main effects of empathic concern (*F*(1, 26) = 11.16, *p* = .003, η_p_² = 0.30) and third-party action were also significant (*F*(1.50, 39.02) = 61.89, *p* < .001, η_p_² = 0.70). The above results indicate that the findings for anger reduction were robust when rank-transformed scores were used.

# S6. Sensitivity analyses of the time-frequency results

To assess whether the alpha and theta power results were driven by extreme participant-level values, we conducted sensitivity analyses using two approaches. First, we assessed the robustness of the observed effects using robust statistical methods based on 20% trimmed means, in which the lowest and highest 20% of observations are omitted when estimating the mean. Such methods are less sensitive to extreme observations and departures from normality than conventional mean-based methods (Erceg-Hurn & Mirosevich, 2008; Field & Wilcox, 2017; Mair & Wilcox, 2020; Wilcox, 1998). Second, following the ± 3 *SD* exclusion procedures used in previous social decision-making studies (Hang et al., 2026; Martiny-Huenger et al., 2021; Y. Zhang et al., 2025), we identified extreme values using the mean ± 3 *SD* criterion and repeated the original analyses after excluding the corresponding participants.

## S6.1. Robust analyses based on 20% trimmed means

### S6.1.1 Method

The robust analyses based on 20% trimmed means were implemented using the WRS2 package in R version 4.4.2 (Mair & Wilcox, 2020). For theta power, because the original finding concerned the main effect of third-party action, theta power was averaged across the empathy and non-empathy conditions separately for no-punishment outcomes, partial-punishment outcomes, and complete-punishment outcomes. These three within-participant conditions were analyzed using a robust repeated-measures ANOVA based on 20% trimmed means, followed by robust multiple comparisons (Mair & Wilcox, 2020).

For alpha power, because the original finding concerned the main effect of empathic concern, alpha power was averaged across the three third-party actions separately for the empathy and non-empathy conditions. The two resulting within-participant conditions were compared using Yuen’s robust test for dependent samples based on 20% trimmed means (Mair & Wilcox, 2020).

### S6.1.2 Results

**Theta.** A robust repeated-measures ANOVA based on 20% trimmed means revealed a significant main effect of third-party action, *F*(2, 32) = 4.33, *p* = .022. The 20% trimmed means were 0.21 for no-punishment outcomes, 0.33 for partial-punishment outcomes, and 0.18 for complete-punishment outcomes. Robust multiple comparisons showed greater theta power for partial-punishment outcomes than for complete-punishment outcomes (estimated difference = 0.130, 95% CI [0.011, 0.248], *p* = .010). Neither the comparison between no-punishment and partial-punishment outcomes (*p* = .170) nor that between no-punishment and complete-punishment outcomes (*p* = .698) was significant.

**Alpha.** Yuen’s robust test for dependent samples revealed a significant difference between the empathy condition (20% trimmed *M* = −0.15) and the non-empathy condition (20% trimmed *M* = −0.23), *t*(16) = 3.01, *p* = .008, trimmed-mean difference = 0.081, 95% CI [0.024, 0.139].

Overall, the 20% trimmed-mean analyses supported both the third-party action effect observed in theta power and the empathic concern effect observed in alpha power.

## S6.2. Sensitivity analyses based on the mean ± 3 SD criterion

### S6.2.1 Method

First, we conducted a condition-wise outlier analysis. The mean and *SD* of alpha and theta power were calculated across participants separately for each of the six experimental conditions. Values falling outside the corresponding condition mean ± 3 *SD* were identified as extreme values. Participants with at least one value meeting this criterion were excluded, and the original analyses were repeated.

Second, we conducted effect-specific outlier analyses based on within-participant difference scores. For theta power, we first averaged theta power across the empathy and non-empathy conditions separately for each third-party action outcome. We then calculated three within-participant difference scores, including theta power induced by partial-punishment outcomes minus that induced by complete-punishment outcomes (PP–CP), theta power induced by partial-punishment outcomes minus that induced by no-punishment outcomes (PP–NP), and theta power induced by no-punishment outcomes minus that induced by complete-punishment outcomes (NP–CP). Each difference score was evaluated using its corresponding sample mean ± 3 *SD*. For alpha power, we first averaged alpha power across the three third-party actions separately for the empathy and non-empathy conditions. For each participant, the difference between alpha power in the empathy condition and that in the non-empathy condition (Empathy minus Non-empathy) was then calculated. Participants whose difference scores fell outside this criterion were excluded, and the original analyses were repeated.

### S6.2.2 Results

**Theta.** First, in the condition-wise analysis, three participants exceeded the ± 3 *SD* criterion (Fig. S3a). After excluding these participants, the main effect of third-party action was not significant (*F*(2, 46) = 2.81, *p* = .071, η_p_² = 0.11). Theta power did not differ significantly among no-punishment outcomes (0.22 ± 0.36), partial-punishment outcomes (0.31 ± 0.32), and complete-punishment outcomes (0.19 ± 0.25; *p*s > .05).

Second, in the effect-specific analysis, one participant’s NP–CP theta power difference fell outside the corresponding sample mean ± 3 *SD* (Fig. S3b), whereas no participant exceeded the criterion for either the PP–CP or PP–NP difference. After excluding this participant, the main effect of third-party action was not significant (*F*(2, 50) = 2.92, *p* = .063, η_p_² = 0.10). Theta power did not differ significantly among no-punishment outcomes (0.30 ± 0.46), partial-punishment outcomes (0.38 ± 0.40), and complete-punishment outcomes (0.26 ± 0.39; *p*s > .05).

**Alpha.** First, in the condition-wise analysis, one participant exceeded the ± 3 *SD* criterion in all six alpha power conditions (Fig. S3c). After excluding this participant, the main effect of empathic concern remained significant, *F*(1, 25) = 4.91, *p* = .036, η_p_² = 0.16. Alpha power was lower in the non-empathy condition (−0.37 ± 1.13) than in the empathy condition (−0.30 ± 1.12), *p* = .036, Cohen’s *d* = −0.43. No other main effects or interactions were significant.

Second, in the effect-specific analysis, no participant’s Empathy minus Non-empathy alpha power difference fell outside the sample mean ± 3 *SD* (Fig. S3d). Thus, no participant was excluded on the basis of the effect-specific criterion.

**Fig. S3. Outlier inspection of theta and alpha power across experimental conditions and effect-specific difference scores. a,** Theta power across the six experimental conditions. **b,** Within-participant theta power differences between third-party action conditions (PP–CP, PP–NP, and NP–CP). Black diamonds indicate the mean for each condition or difference score. **c,** Alpha power across the six experimental conditions. **d,** Within-participant alpha power differences between the empathy and non-empathy conditions (Empathy minus Non-empathy). Circles represent individual participants’ values, with gray circles indicating values within ± 3 *SD* of the corresponding mean and colored circles indicating values exceeding ± 3 *SD*, which were considered potential outliers. The same color denotes the same participant. Dashed horizontal lines indicate the upper and lower boundaries of the corresponding mean ± 3 *SD*. *Note.* NP = no punishment; PP = partial punishment; CP = complete punishment; PP–CP = partial-punishment outcomes minus complete-punishment outcomes; PP–NP = partial-punishment outcomes minus no-punishment outcomes; NP–CP = no-punishment outcomes minus complete-punishment outcomes.

Overall, the exclusion-based analyses indicated that the effect observed in the alpha band was relatively stable under the ± 3 *SD* criterion, whereas the effect observed in the theta band was more sensitive to the exclusion of participants with extreme values.

Taken together, the empathic concern effect observed in alpha power was consistently supported across both sensitivity analyses. For theta power, the 20% trimmed-mean robust analysis retained both the main effect of third-party action and the difference between partial-punishment and complete-punishment outcomes, whereas the main effect of third-party action did not remain significant after excluding participants identified by the ± 3 *SD* criterion. These findings suggest that the effect of third-party action observed in theta power is more sensitive to the treatment of extreme values. Accordingly, we have qualified the interpretation of the theta finding in the *General discussion* and note that its stability should be further evaluated in future studies with larger samples.

# References

Erceg-Hurn, D. M., & Mirosevich, V. M. (2008). Modern robust statistical methods: An easy way to maximize the accuracy and power of your research. *American Psychologist, 63*(7), 591–601. https://doi.org/10.1037/0003-066X.63.7.591

Evans, J. S. B. T. (2003). In two minds: Dual-process accounts of reasoning. *Trends in Cognitive Sciences*, *7*(10), 454–459. https://doi.org/10.1016/j.tics.2003.08.012

Evans, J. S. B., & Stanovich, K. E. (2013). Dual-process theories of higher cognition: Advancing the debate. *Perspectives on Psychological Science*, *8*(3), 223–241. https://doi.org/10.1177/1745691612460685

Feng, C., Luo, Y. J., & Krueger, F. (2015). Neural signatures of fairness-related normative decision making in the ultimatum game: A coordinate-based meta-analysis. *Human Brain Mapping*, *36*(2), 591–602. https://doi.org/10.1002/hbm.22649

Field, A. P., & Wilcox, R. R. (2017). Robust statistical methods: A primer for clinical psychology and experimental psychopathology researchers. *Behaviour Research and Therapy*, *98*, 19–38. https://doi.org/10.1016/j.brat.2017.05.013

Hang, Y., Wu, W., Li, R., Huang, L., Shioiri, S., & He, X. (2026). Trust emerges from shared attention: Behavioural and neural evidence from dual EEG hyperscanning. *Human Brain Mapping*, *47*(4), e70504. https://doi.org/10.1002/hbm.70504

Hayes, A. F. (2018). *Introduction to mediation, moderation, and conditional process analysis: A regression-based approach* (2nd ed.). Guilford Press.

Mair, P., & Wilcox, R. (2020). Robust statistical methods in R using the WRS2 package. *Behavior Research Methods*, *52*(2), 464–488. https://doi.org/10.3758/s13428-019-01246-w

Martiny-Huenger, T., Bieleke, M., Doerflinger, J., Stephensen, M. B., & Gollwitzer, P. M. (2021). Deliberation decreases the likelihood of expressing dominant responses. *Psychonomic Bulletin & Review*, *28*(1), 139–157. https://doi.org/10.3758/s13423-020-01795-8

Mendoza, S. A., Lane, S. P., & Amodio, D. M. (2014). For members only: Ingroup punishment of fairness norm violations in the ultimatum game. *Social Psychological and Personality Science*, *5*(6), 662–670. https://doi.org/10.1177/1948550614527115

Ochsner, K. N., & Gross, J. J. (2005). The cognitive control of emotion. *Trends in Cognitive Sciences*, *9*(5), 242–249. https://doi.org/10.1016/j.tics.2005.03.010

Ochsner, K. N., Ray, R. R., Hughes, B., McRae, K., Cooper, J. C., Weber, J., ... & Gross, J. J. (2009). Bottom-up and top-down processes in emotion generation: common and distinct neural mechanisms. *Psychological Science*, *20*(11), 1322–1331. https://doi.org/10.1111/j.1467-9280.2009.0245

Schwarz, N., & Clore, G. L. (1983). Mood, misattribution, and judgments of well-being: Informative and directive functions of affective states. *Journal of Personality and Social Psychology*, *45*(3), 513–523. https://doi.org/10.1037/0022-3514.45.3.513

Strack, F., & Deutsch, R. (2004). Reflective and impulsive determinants of social behavior. *Personality and Social Psychology Review*, *8*(3), 220–247. https://doi.org/10.1207/s15327957pspr0803_1

Wilcox, R. R. (1998). How many discoveries have been lost by ignoring modern statistical methods? *American Psychologist*, *53*(3), 300–314. http://dx.doi.org/10.1037//0003-066x.53.3.300.

Zhang, Y. X., Dong, J., Chen, N., & Wei, P. (2025). Effort expenditure reduces prosocial decision‐making: Computational principles and neural mechanisms. *Human Brain Mapping*, *46*(11), e70290. https://doi.org/10.1002/hbm.70290

Zhang, Z., Li, M., Liu, Q., Chen, C., & Qi, C. (2023). Group membership and adolescents’ third-party punishment: A moderated chain mediation model. *Frontiers in Psychology*, *14*, 1251276. https://doi.org/10.3389/fpsyg.2023.1251276
